# Socioeconomic factors and inequality in the distribution of physicians and nurses in Mexico

**DOI:** 10.11606/s1518-8787.2020054002011

**Published:** 2020-06-03

**Authors:** Julio César Montañez-Hernández, Jacqueline Alcalde-Rabanal, Hortensia Reyes-Morales

**Affiliations:** I Instituto Nacional de Salud Pública Centro de Investigación en Sistemas de Salud CuernavacaMor México Instituto Nacional de Salud Pública. Centro de Investigación en Sistemas de Salud. Cuernavaca, Mor, México

**Keywords:** Distribution of Physicians, Nurses, provision & distribution, Patient Care Group, Socio-Economic Factors, Equity in Health

## Abstract

**OBJECTIVE:**

To describe the human resources for health and analyze the inequality in its distribution in Mexico.

**METHODS:**

Cross-sectional study based on the National Occupation and Employment Survey (ENOE in Spanish) for the fourth quarter of 2018 in Mexico. Graduated physicians and nurses, and auxiliary/technician nurses with completed studies were considered as human resources for health. States were grouped by degree of marginalization. Densities of human resources for health per 1,000 inhabitants, Index of Dissimilarity (DI) and Concentration Indices (CI) were estimated as measures of unequal distribution.

**RESULTS:**

The density of human resources for health was 4.6 per 1,000 inhabitants. We found heterogeneity among states with densities from 2.3 to 10.5 per 1,000 inhabitants. Inequality was higher in the states with a very low degree of marginalization (CI = 0.4) than those with high marginalization (CI = 0.1), and the inequality in the distribution of physicians (CI = 0.5) was greater than in graduated nurses (CI = 0.3) among states. In addition, 17 states showed a density above the threshold of 4.5 per 1,000 inhabitants proposed in the Global Strategy on Human Resources for Health. That implies a deficit of nearly 60,000 human resources for health among the 15 states below the threshold. For all states, to reach a density equal to the national density of 4.6, about 12.6% of human health resources would have to be distributed among states that were below national density.

**CONCLUSIONS:**

In Mexico, there is inequality in the distribution of human resources for health, with state differences. Government mechanisms could support the balance in the labor market of physicians and nurses through a human resources policy.

## INTRODUCTION

Human resources working in health institutions are considered one of the fundamental components for the proper performance of health systems^1,2^. Anand and Bärnighausen criticize the conceptual framework of health systems proposed by the World Health Organization (WHO), which considers human resources for health (HRH) as one of the building blocks of the system^1^. They propose a conceptual framework in which human resources are the core of health systems^3^. The fulfillment of goals and patient satisfaction depend on the HRH, since they provide preventive and curative care, as well as information on diagnosis, treatment and monitoring, and decide which technology and/or drug to use. Therefore, their size, composition and distribution are very relevant to ensure the population’s access to health services^3^.

The availability of HRH, measured by density per number of inhabitants, has been used as a health coverage indicator. A positive relationship has been found with some interventions, such as vaccination or births performed by qualified personnel^4^. In 2004, the Joint Learning Initiative, [JLI] recommended a density of 2.5 per 1,000 inhabitants to achieve 80.0% measles immunization coverage and deliveries performed by health personnel^5^. Under the Millennium Development Goals (MDG), the 2006 WHO Global Report suggested a minimum threshold of 2.3 physicians, nurses and midwives per thousand inhabitants to reach 80.0% of deliveries performed by qualified personnel; 57 countries did not reach such an indicator^4^.

In 2016, under the Sustainable Development Goals (SDG)^6^, the WHO established a density of 4.5 physicians, nurses and midwives per 1,000 inhabitants as the minimum threshold required for the implementation of the Global Strategy on Human Resources for Health (GSHRH) under Universal Health Coverage (UHC)^7^. Unlike the 2006 indicator, this threshold includes 12 indicators related to UHC and SDG targets. With this indicator, the WHO predicted that 18 million more health workers would be needed in low-middle-income countries to achieve SDG^7,8^, although it became an emphasis that it should not be a target for all countries given their differences in health needs^7^.

In Mexico, the federal government launched the public health insurance known as *Seguro Popular de Salud* (PHI – Popular Health Insurance) in 2003, to provide financial protection to the population excluded from social security^9^. At the end of 2017, 53.5 million Mexicans were affiliated with PHI^10^. In 2005–2016, public spending on health per capita increased by 31.0%, and the density of physicians and nurses per thousand inhabitants increased by 34.0 and 32.0%, respectively. Despite these advances, the public health system faces problems of accessibility and quality in care, which can be explained by the lack of an explicit policy on human resources for health^11^ and by the imbalance between the health needs of the population and human and financial resources^11,12^.

At national level, the *Secretaría del Trabajo y Previsión Social* (Ministry of Labor and Social Welfare) makes periodic estimates of the number of graduated physicians and nurses who are employed^13^; however, these estimates do not show whether employed professionals practice the medical profession nor specify the length of the working day. Other sources of information such as the Organization for Economic Cooperation and Development (OECD) indicate for Mexico a density of 2.4 and 2.9 physicians and nurses, respectively, in 2017. However, these data include intern physicians (undergraduate) and residents (in graduate specialization), use different sources of information and recognize the possibility of duplication^14^. In both cases, the HRH density indicator may be overestimated, and it is not possible to estimate the professional deficit that is necessary to reach the 4.5 threshold recommended in GSHRH. Therefore, this study aimed to describe the HRH and analyze it’s inequal distribution in Mexico.

## METHODS

Cross-sectional study based on secondary data analysis to describe characteristics of the physicians and nurses. The National Occupation and Employment Survey (ENOE, in Spanish), conducted by the National Institute of Statistics and Geography (INEGI, in Spanish) was used. ENOE has a probabilistic, two-phased, stratified and clustered design. It is carried out quarterly, has national and state geographic coverage, and it aims to provide information on the occupational characteristics of the population aged 15 and older^15^. Databases are publicly available and do not contain identifiable information about individuals.

ENOE-based inequality indicators were constructed for the fourth quarter of 2018 (quarter IV-2018). All professionals with medical or nursing studies who reported completing their undergraduate studies at the time of the survey were considered Human Resources for Health (HRH) or health workers. We also included those who studied nursing at the technical level (auxiliary nursing).

### Variables16,17

Employed (Emp): physicians and nurses who worked 20 hours or more a week performing functions according to their proffesional training in the health area, or performing administrative functions in the health sector.

Unemployed (Une): physicians and nurses who looked for a job because they did not have an economic activity.

Quantitative underemployment (QuantiUn): physicians and nurses employed less than 20 hours a week and with functions according to their profession, or who exercised the health profession as a secondary job.

Qualitative underemployment (QualiUn): physicians and nurses employed but with functions or activities outside their professional training, regardless of working hours per week.

Economically Active Population (EAP): physicians and nurses employed, underemployed and unemployed.

### Inequality Measures

Densities of Human Resources for Health per 1,000 inhabitants:

a.Density of employed human resources (DHRH): $\frac{Emp}{Total\;Population}\;x\;1.000$.

b.Density of Human Resources EAP: $\frac{Emp\;+\;\mathrm{QauntiUn}\;+\;\mathrm{QualiUn}\;+\;\mathrm{Une}}{Total\;Population}\;x\;1.000$.

In both cases, the HRH deficit was obtained from the threshold recommended in GSHRH according to the following formulas:

c.Employed deficit: $\frac{(4,5–\mathrm{Density}\;\mathrm{of}\;\mathrm{employed}\;\mathrm{human}\;\mathrm{resources})\;x\;\mathrm{total}\;\mathrm{population}}{1.000}\;$.

d.EAP Deficit: $\frac{(4,5–\mathrm{Density}\;\mathrm{of}\;\mathrm{Human}\;\mathrm{Resources}\;\mathrm{EAP})\;x\;\mathrm{total}\;\mathrm{population}}{1.000}\;$.

Socio-economic factors (age, schooling, residence and characteristics of employment) in both professions were analyzed, and state densities of human resources for health of 1) employed health personnel per 1,000 inhabitants (DHRH), and 2) economically active population (EAP) per 1,000 inhabitants were estimated. In both cases, densities were compared with the density of 4.5 per 1,000 inhabitants recommended in GSHRH, and the number of health workers needed to meet this threshold was estimated.

### Index of dissimilarity

The Index of dissimilarity (ID) is one indicator used in the analysis of health inequalities^18,19^. It is estimated using the formula $\sum_{j=1}^{32}\;\frac{1}{2}\;\left|s_{jh}\;-\;s_{jp}\right|$, where *s*_*jh*_ is the proportion of HRH in state *j* with respect to the national total of HRH, and *s*_*jp*_ is the proportion of the population in state *j* with respect to the national population. In this study, the ID was interpreted as the proportion (or percentage) of workers who would have to be redistributed among states so that everyone had the same DHRH.

### Index and concentration curve

Another index used in the analysis of health inequalities is the index from the Concentration Curve (CC)^18,19^. In this study, curves were elaborated considering states as an analysis unit. The proportion of HRH in state j with respect to the national total of HRH (*s*_*jh*_) was estimated, and the proportion of Disability-Adjusted Life Year (DALY) in state *j* was estimated from the national total of DALYs (*s*_*jd*_). The concentration curve plots the cumulative proportion of DALY by the states (starting with the state with the lowest proportion of DALY on the X-axis, and ending with the highest) with the accumulated proportions of HRH in the Y-axis. If workers are equally distributed among states according to their percentage of the national disease burden, the concentration curve matches the diagonal joining the points (0.0) and (1.1), which is called the equality line. The area between the CC and the equality line is the Concentration Index (CI) and the higher the absolute value of the larger IC, higher the inequality. A scatter plot was built between the DHRH per 1,000 inhabitants and the DALY per 100 thousand inhabitants in each state^18^. In addition, another dispersion plot was built between the DHRH per 1,000 inhabitants and the Gross Domestic Product (GDP) per capita, and the Spearman’s correlation was estimated.

### Analysis

The three inequality indicators in the distribution of human resources at the national level and by states grouped by degree of marginalization were used: the DHRH per 1,000 inhabitants, the ID and the CI. Population size information comes from the population projections of the National Population Council (CONAPO, in Spanish) available on the website of the Directorate-General for Health Information (DGIS, in Spanish) of the Ministry of Health^20^. The classification and grouping of states according to degree of marginalization comes from the classification proposed by CONAPO^21^. Data on the Gross Domestic Product (GDP) were obtained from the INEGI website^22^. DALY information was obtained from global disease burden estimates conducted by the Institute for Health Metrics and Evaluation (IHME)^23^.

The analyses were conducted using the survey weights, and its complex design was considered using the SVY module of the STATA MP 13.0 package. Pearson Chi-square tests assess the differences in distribution between the type of profession (medicine or nursing) and its socioeconomic factors considering p < 0.05 for statistical significance and confidence intervals at 95% (95%CI).

## RESULTS

In 2018, there were 413,000 physicians and 714,000 nurses in Mexico, of whom 62.9% and 44.4%, respectively, were employed 20 hours or more in the health sector (Table 1), which is equivalent to 260,482 physicians and 317,280 nurses (Table 2); 24.8% of physicians [95%CI 20.7–29.3] and 1.5% of nurses had a specialty [95%CI 1.0–2.2]. By contrast, 46.8% [95%CI 44.1–49.6] were auxiliary nurses/technicians. We found statistically significant differences in age distribution and employment characteristics in both professions. In particular, qualitative underemployment was higher in nursing personnel (17.6%, [95%CI 15.3–20.2]) than in medical staff (8.8%, [95%CI 6.9–11.0]), as well as unemployment (1.9%, [95%CI 1.2–2.9]; and 1.3%, [95%CI: 0.7–2.4], respectively) (Table 1).

**Table 1:** Socioeconomic factors of medical and nursing staff. National Occupation and Employment Survey (ENOE in Spanish), Mexico, Quarter IV, 2018.

	Total	Medical Staff	Nursing Staff
N	1,128,668	413,866	714,802
n	4,022	1,443	2,579
%	100%	36.7%	63.3%
	**% [95%CI]**		
**Age group (years)**			
20–29	21.1 [19.3–23.0]	16.6 [13.8–19.8]	23.7 [21.5–26.0]
30–39	24.4 [22.2–26.8]	26.5 [22.3–31.1]	23.2 [20.7–25.9]
40–49	17.5 [15.7–19.4]	14.0 [11.4–17.0]	19.5 [17.2–22.0]
50–59	18.8 [16.8–21.0]	15.8 [12.8–19.3]	20.6 [18.1–23.4]
60–69	13.0 [11.2–15.0]	19.0 [15.5–23.1]	9.5 [7.8–11.4]
70+	5.2 [4.1–6.5]	8.1 [5.9–11]	3.5 [2.6–4.7]
**Schooling**			
Auxiliary/Technicians	29.7 [27.4–32.0]		46.8 [44.1–49.6]
Bachelor degree	60.3 [57.7–62.9]	75.2 [70.7–79.3]	51.7 [48.9–54.5]
Specialty	10.0 [8.4–11.9]	24.8 [20.7–29.3]	1.5 [1.0–2.2]
**Marital status**			
Single^b^	40.6 [38.1–43.1]	40.0 [35.8–44.3]	40.9 [38.0–44.0]
Has a partner^c^	59.4 [56.9–61.9]	60.0 [55.7–64.2]	59.1 [56.0–62.0]
**Employment characteristics**			
EAP			
Employment	51.2 [48.6–53.8]	62.9 [58.8–66.9]	44.4 [41.3–47.5]
Quantitative underemployment	4.2 [3.3–5.3]	5.7 [4.0–8.0]	3.3 [2.4–4.5]
Qualitative underemployment	14.3 [12.7–16.2]	8.8 [6.9–11.0]	17.6 [15.3–20.2]
Underemployment	1.7 [1.2–2.4]	1.3 [0.7–2.4]	1.9 [1.2–2.9]
EIP^d^	28.6 [26.3–31.0]	21.3 [18.1–24.8]	32.8 [29.9–35.9]
**Residence place**			
Rural	6.1 [5.6–6.8]	4.7 [4.3–5.1]	7.0 [6.4–7.7]
Urban	93.9 [93.2–94.4]	95.3 [94.9–95.7]	93[92.3–93.6]
**Degree of marginalization**^e^			
Very low	24.3 [23.0–25.7]	27.3 [24.8–30.0]	22.6 [21.3–24.0]
Low	32.0 [30.8–33.2]	31.1 [29.3–33.1]	32.4 [31.1–33.7]
Medium	14.2 [13.6–14.9]	13.6 [12.6–14.6]	14.6 [13.9–15.3]
High	21.7 [20.7–22.8]	20.0 [18.9–21.1]	22.7 [21.5–23.9]
Very high	7.8 [7.3–8.4]	8.0 [7.3–8.7]	7.7 [7.1–8.3]

ENOE: National Occupation and Employment Survey; EAP: Economically Active Population; EIP Economically Inactive Population

^a^ p < 0.000; ^b^ Includes singles, divorcees and widowers; ^c^ Includes united and married; ^d^ Economically Inactive Population: includes people engaged in household activities, students, pensioners and the disabled. ^e^ **Degree of marginalization**: Mexico City, Coahuila, Baja California, Nuevo León **(Very low marginalization)**; Aguascalientes, Baja California Sur, Colima, Chihuahua, Jalisco, State of Mexico, Tamaulipas **(Low marginalization)**; Durango, Guanajuato, Morelos, Nayarit, Querétaro, Quintana Roo, Sonora, Tlaxcala, Zacatecas **(Moderate marginalization)**; Campeche, Hidalgo, Michoacán, Puebla, San Luis Potosí, Sinaloa, Tabasco, Veracruz, Yucatán (High marginalization); Chiapas, Guerrero, Oaxaca **(Very high marginalization)**.

**Table 2:** Measures of inequality in the distribution of employed medical and nursing staff in states grouped by degree of marginalization: Density per 1,000 inhabitants (DHRH), Index of dissimilarity (ID) and Concentration Index (CI). National Occupation and Employment Survey (ENOE in Spanish), Mexico, Quarter IV, 2018.

	National (32)	Degree of marginalization (number of states)				

		Very low (4)	Low (7)	Moderate (9)	High (9)	Very High (3)
Population (thousands)	124,738	20,786	39,261	20,849	30,687	13,155
Total HRH (medicine and nursing)	577,762	150,807	173,821	83,027	124,337	45,770
DHRH	4.6	7.3	4.4	4.0	4.0	3.5
ID	12.6	18.9	6.6	7.7	11.5	2.7
CI	0.4	0.4	0.5	0.2	0.2	0.1
Physicians	260,482	80,511	77,279	33,587	49,861	19,244
DHRH	2.1	3.9	2.0	1.6	1.6	1.5
ID	16.3	30.0	6.5	7.1	10.6	5.5
CI	0.5	0.5	0.5	0.3	0.2	0.0
Technical nursing	111,856	25,811	46,507	11,562	25,051	2,925
DHRH	0.9	1.2	1.2	0.5	0.8	0.2
ID	25.4	8.6	9.4	13.4	33.6	29.0
CI	0.5	0.3	0.6	0.3	0.2	0.2
Professional nursing	205,424	44,485	50,035	37 878	49,425	23,601
DHRH	1.6	2.1	1.3	1.8	1.6	1.8
ID	18.2	12.1	26.1	12.4	15.4	6.1
CI	0.3	0.3	0.3	0.2	0.2	0.1

DHRH: Density of employed Human Resources for Health per 1,000 inhabitants. ID: Index of dissimilarity. CI: Concentration Index.

The density of health professionals (DHRH) per 1,000 inhabitants was 4.6 (2.1 in medical staff, 0.9 and 1.6 in technicians and professional nurses, respectively), exceeding the threshold of 4.5 recommended in GSHRH (Table 2). However, the gap between the current density and the 4.5 threshold was heterogeneous among states according to degree of marginalization: while in very low-grade states the density is 7.3 per 1,000 inhabitants, in the very high-marginalization group was 3.5 per 1,000 inhabitants. The ID showed that, in order for all states to have a density of 4.6 per 1,000 inhabitants, 12.6% of the total health workforce would have to be redistributed, while, in the group of states of very high marginalization, 2.7% would have to be distributed so that everyone would reach a density of 3.5 per 1,000 inhabitants. In addition, the national CI (0.4) showed similar inequality at the national level as within the very low and low marginalization groups (0.4 and 0.5, respectively), and inequality was similar in the moderate- and high-grade groups (0.2 and 0.2, respectively), although lower than in the previous groups.

**Figure 1:**
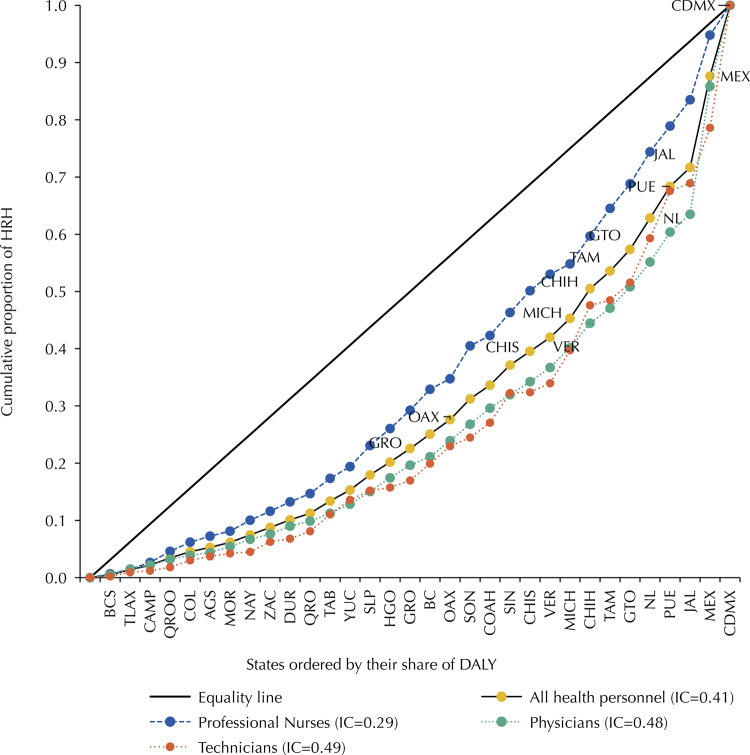
Employed Human Resources for Health (HRH) concentration curve used among states ordered according to the proportion of Disability-Adjusted Life Year, DALY. National Occupation and Employment Survey (ENOE, in Spanish), Mexico, Quarter IV, 2018. Aguascalientes (AGS), Baja California (BC), Baja California Sur (BCS), Campeche (CAMP), Coahuila (COAH), Colima (COL), Chiapas (CHIS), Chihuahua (CHIH), Mexico City (CDMX), Durango (DGO), Guanajuato (GTO), Guerrero (GRO), Hidalgo (HGO), Jalisco (JAL), State of México (MEX), Michoacán (MICH), Morelos (MOR), Nayarit (NAY), Nuevo León (NL), Oaxaca (OAX), Puebla (PUE), Querétaro (QRO), Quintana Roo (QROO), San Luis Potosí (SLP), Sinaloa (SIN), Sonora (SON), Tabasco (TAB), Tamaulipas (TAMP), Tlaxcala (TLAX), Veracruz (VER), Yucatán (YUC), Zacatecas (ZAC).

**Figure 2:**
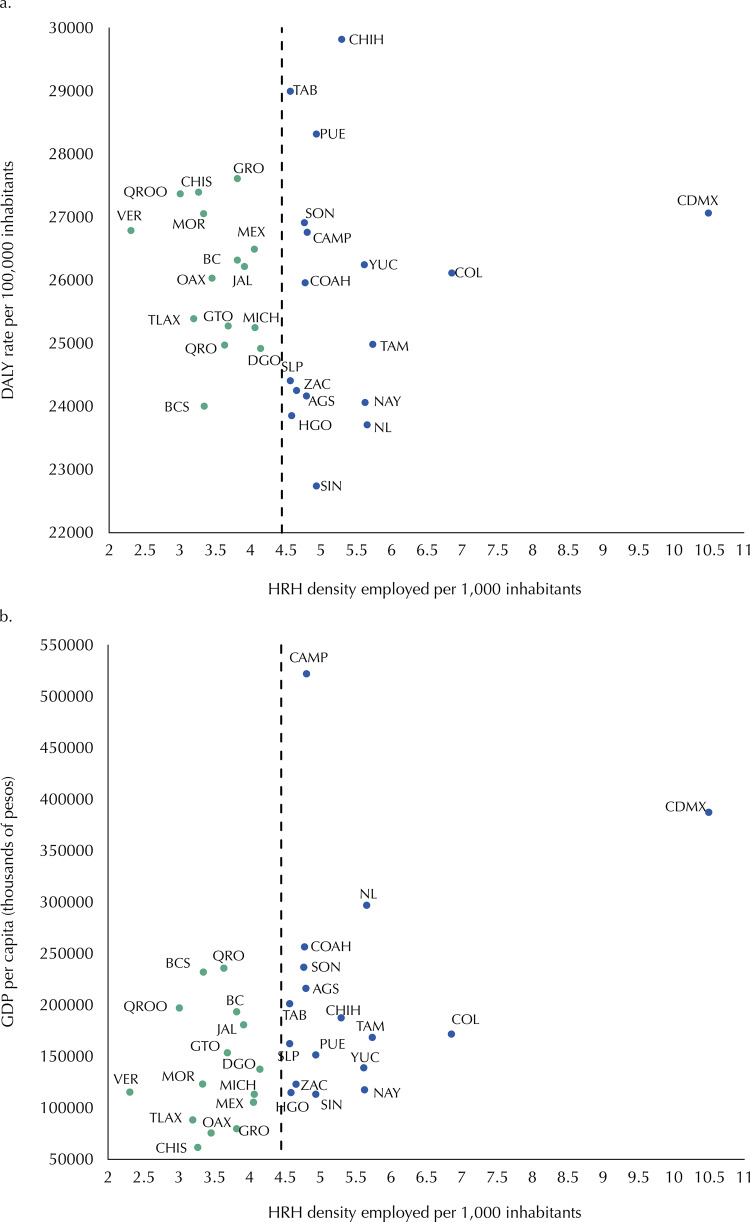
Dispersion between the density of Human Resources for Health (HRH) employed with the rates of Disability-Adjusted Life Year (DALY) per 100,000 inhabitants and with GDP *per capita*. National Occupation and Employment Survey, ENOE, Mexico, Quarter IV, 2018. Aguascalientes (AGS), Baja California (BC), Baja California Sur (BCS), Campeche (CAMP), Coahuila (COAH), Colima (COL), Chiapas (CHIS), Chihuahua (CHIH), Mexico City (CDMX), Durango (DGO), Guanajuato (GTO), Guerrero (GRO), Hidalgo (HGO), Jalisco (JAL), State of México (MEX), Michoacán (MICH), Morelos (MOR), Nayarit (NAY), Nuevo León (NL), Oaxaca (OAX), Puebla (PUE), Querétaro (QRO), Quintana Roo (QROO), San Luis Potosí (SLP), Sinaloa (SIN), Sonora (SON), Tabasco (TAB), Tamaulipas (TAMP), Tlaxcala (TLAX), Veracruz (VER), Yucatán (YUC), Zacatecas (ZAC).

Half of the states (50.0%) with the smallest proportions of DALY concentrated less than 25.0% of the entire workforce, while five of them (Nuevo León, Puebla, Jalisco, Mexico and Mexico City) concentrated more than 40.0% (CI = 0.4). This pattern was mainly caused by the inequality in the distribution of medical personnel (CI = 0.5) and technical nursing (CI = 0.5), and to a lesser extent to the distribution of professional nursing (CI = 0.3) (Figure 1). In addition, no correspondence was observed between the DHRH per 1,000 inhabitants in each state and its rate of DALY per 100,000 inhabitants; Veracruz and Mexico City had similar rates but showed the lowest and highest DHRH, respectively. In contrast, Chihuahua and Sinaloa showed DHRH above 4.5 and the highest and lowest rate of DALY, respectively (Figure 2a). On the other hand, a positive correlation was found between the DHRH per 1,000 inhabitants and the Gross Domestic Product (GDP) *per capita* (rho = 0.3718, p < 0.05). This coincided with what was described above, according to which the groups of entities of very low and very high marginalization had higher and lower DHRH, respectively (Figure 2b).

**Figure 3:**
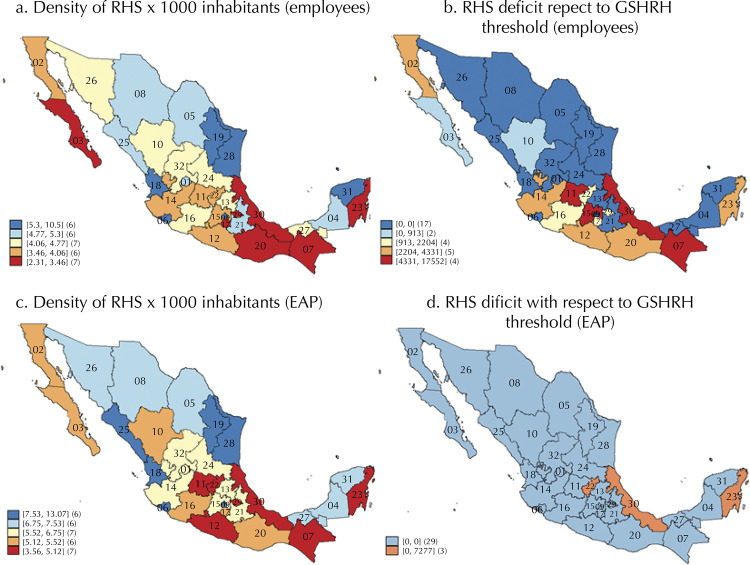
Density of Human Resources in Health (DHRH): employees and Economically Active Population (EAP) per 1,000 inhabitants, and their deficits to reach the threshold of the Global Strategy on Human Resources for Health (GSHRH). National Occupation and Employment Survey (ENOE, in Spanish), Mexico, Quarter IV, 2018. Aguascalientes (01), Baja California (02), Baja California Sur (03), Campeche (04),Coahuila (05), Colima (06), Chiapas (07), Chihuahua (08), Mexico City (09), Durango (10), Guanajuato (11), Guerrero (12), Hidalgo (13), Jalisco (14), State of México (15), Michoacán (16), Morelos (17), Nayarit (18), Nuevo León (19), Oaxaca (20),Puebla (21), Querétaro (22), Quintana Roo (23), San Luis Potosí (24), Sinaloa (25), Sonora (26), Tabasco (27), Tamaulipas (28),Tlaxcala (29),Veracruz (30), Yucatán (31), Zacatecas (32).

Seventeen states reached a density of 4.5 (Figure 2, 3a and 3b). The estimate of the gap in the number of workers to reach the threshold shows that 59,618 workers employed 20 hours or more per week in the health sector would be required for the remaining 15 states to reach the threshold. However, the gap ranged from 4,332 to 17,552 (Figure 3b) among only four of them (Guanajuato, Chiapas, Veracruz and the state of Mexico). On the other hand, in the scenario where the entire economically active population (EAP) of HRH was employed 20 or more hours in the health sector, the national density would be 6.5, although three states would not reach the threshold yet (Figure 3c and 3d).

## DISCUSSION

Our study shows a disadvantage of Mexico in the availability of HRH compared with the average of OECD countries^24^. While, according to GSHRH criteria, the density of health workers could be considered acceptable, inequality within the country is reflected in the variability among states, particularly those with high marginalization that do not reach the threshold. Considering that OECD data include interns and residents, and there is the possibility of double counting of physicians and nurses working simultaneously in the public and private sector, the availability of HRH for lower-density areas is likely to be below current demand and with a larger deficit for future years. In this sense, requirement projections by medical specialists in Mexico have estimated that health needs arising from population aging will require more specialists in internal medicine and surgery than in pediatrics, which will imply a growing challenge for the provision of services^25^.

A relevant finding was the lack of consistency in the relation between the density of health workers and DALY rates. As has been documented in other studies, a negative correlation would be expected, which would support evidence of having more health professionals to reduce the burden of disease^26^. Our results could reflect other weak areas of the health system itself, such as insufficient equipment or required inputs, poor quality of care, as well as conditions typical of the demographic and epidemiological transition. The consequences of these multiple factors are chronic-degenerative health needs that lead to disability for a greater proportion of the population^27^.

By contrast, the positive relationship between DHRH and state GDP could be explained by the following reasons: 1) qualified medical personnel can switch residency to Mexican regions with attractive cities for medical mobility^28^, due to their high economic development, which provide better job opportunities and better income expectations^29^; or 2) the distribution of the number of students enrolled at universities across the country where six states, of very low or low marginalization, concentrate to 50.0% and 35.0% of the enrollments of medical and nursing students in the 2017–2018 school year, respectively; in contrast, three very high-marginalization entities account for 15.7% of registered nursing students and 6.5% of medical students^30^. These imbalances in the distribution of human resources pose a challenge to the health system as the population living in areas of high degree of marginalization is less likely to have access to health services.

Limitations of this analysis are the difficulties in identifying labor mobility, which may affect the estimation of state densities over time, and limitations from ENOE: a) it is not possible to distinguish between levels of care or between insurance schemes, which prevents more precise inequalities among population groups or health needs; b) midwives were not included, since it is not possible to identify them in the survey, nor were other categories of health personnel such as dentists, pharmacists, laboratory technicians or community promoters, who are also considered a health workforce^31^, but are not considered in the estimation of the 4.5 per 1,000 Inhabitants threshold; c) the estimation of employment rates does not include physicians and nurses in health teaching and research activities. Therefore, they may have included as underemployed, and 5) ENOE is a national and state representative survey of the entire population over 15 years; medical and nursing staff account for less than 2.0% of the population, which could be construed as a weakness in outcomes at the state level. However, given the random design of ENOE and the sample sizes are large for both professions, representativeness can be assumed for this subgroup.

In brief, the results of this study indicate inequality in the distribution of HRH across states, which may potentially be linked to the number of enrollment offered in educational institutions, the preference of health personnel to be placed into areas with better living conditions, and greater availability of sources of work in states with greater health infrastructure. The establishment of a new human resources policy is a priority that, based on the health needs of the population, articulates the training of physicians, nurses, and other health professionals for incorporation into health institutions, considering the areas of greatest demand. In addition, it is necessary to regulate professional practice, which encourages balance in the public and private labor market.

## References

[1] 1. World Health Organization. Everybody’s business: strengthening health systems to improve health outcomes: WHO’s framework for action. Geneva: WHO Health Systems and Services (HSS); 2007 [cited 2019 Jan 24]. Available from: https://www.who.int/healthsystems/strategy/everybodys_business.pdf

[2] 2. Campbell J, Buchan J, Cometto G, David B, Dussault G, Fogstad H, et al. Human resources for health and universal health coverage: fostering equity and effective coverage. Bull World Health Organ. 2013;91(11):853-63. 10.2471/BLT.13.118729 PMC3853950 24347710

[3] 3. Anand S, Bärnighausen T. Health workers at the core of the health system: framework and research issues. Health Policy. 2012;105(2-3):185-91. 10.1016/j.healthpol.2011.10.012 22154420

[4] 4. Organización Mundial de la Salud. El informe sobre la salud en el mundo 2006 – Colaboremos por la salud. Ginebra: OMS; 2006 [cited 2019 Feb 3]. Available from: https://www.who.int/whr/2006/es/

[5] 5. Chen L, Evans T, Anand S, Boufford JY, Brown H, Chowdhury M, et al. Human resources for health: overcoming the crisis. Lancet. 2004;364(9449):1984-90. 10.1016/S0140-6736(04)17482-5 15567015

[6] 6. United Nations. Sustainable Development Goal 3 -Good Health and Wellbeing. New York: UN; 2019. [cited 2019 Jan 18]. Available from: https://www.un.org/sustainabledevelopment/health/

[7] 7. World Health Organization. Global strategy on human resources for health: Workforce 2030. Geneva: WHO; 2016 [cited 2019 Feb 5]. Available from: https://www.who.int/hrh/resources/global_strategy_workforce2030_14_print.pdf?ua=1

[8] 8. Mandeville KL, Lagarde M, Hanson K, Mills A. Human resources for health: time to move out of crisis mode. Lancet. 2016;388(10041):220-2. 10.1016/S0140-6736(16)30952-7 27479555

[9] 9. Gómez-Dantés O, Ortiz M. Seguro Popular de Salud: siete perspectivas. Salud Publica Mex. 2004;46(6):585-8. 10.1590/S0036-36342004000600013 15624862

[10] 10. Secretaría de Salud (MEX), Comisión Nacional de Protección Social en Salud. Sistema de Protección Social en Salud: informe de resultados Enero-Diciembre 2017. México, DF; 2018 [cited 2019 Feb 15]. Available from: http://www.transparencia.seguro-popular.gob.mx/contenidos/archivos/transparencia/planesprogramaseinformes/informes/2017/InformedeResultadosdelSPSSenero-diciembre2017.pdf

[11] 11. Rivera Dommarco J, Pérez Cuevas R, Reyes Morales H, Lazcano Ponce E, Alpuche Aranda C, Shamah Levy T, et al. Salud pública y atención primaria. Base del acceso efectivo a la salud de los mexicanos. Cuernavaca (MEX): Instituto Nacional de Salud Pública; 2018 [cited 2019 Feb 12]. Available from: https://www.insp.mx/images/stories/2018/Docs/180919_Salud_atencion_primaria_11septiembre.pdf

[12] 12. Rivera Dommarco JA, Colchero Aragonés MA, Fuentes ML, González de Cosío Martínez T, Aguilar Salinas CA; Hernández Licona G, et al. La obesidad en México. Estado de la política pública y recomendaciones para su prevención y control. Cuernavaca (MEX): Instituto Nacional de Salud Pública; 2018 [cited 2019 Feb 12]. Available from: https://www.insp.mx/produccion-editorial/novedades-editoriales/4971-obesidad-mexico-politica-publica-prevencion-control.html

[13] 13. Secretaría del Trabajo y Previsión Social (MEX), Servicio Nacional de Empleo: Observatorio Laboral. México, DF; 2020 [cited 2020 Apr 5]. Available from: https://www.observatoriolaboral.gob.mx/static/estudios-publicaciones/Biologia.html

[14] 14. Organisation for Economic Co-Operation and Development. OECD Statistics: Health: Health Care Resources. Paris: OECD; 2019 [cited 2019 Jun 28]. Available from: https://stats.oecd.org/

[15] 15. Instituto Nacional de Estadística y Geografía. Encuesta Nacional de Ocupación y Empleo-ENOE 2018. Aguascalientes (MEX): INEGI 2018 [cited 2018 Apr 23]. Available from: https://www.inegi.org.mx/programas/enoe/15ymas/

[16] 16. Frenk J, Nigenda DG, Munoz-delRio A, Robledo C, Luis A. Patterns of medical employment: a survey of imbalances in urban México. Am J Public Health. 1991;81(1):23-9. 10.2105/ajph.81.1.23 PMC1404928 1983912

[17] 17. Aguilar AM, Nigenda G, Méndez O, Knaul FM. Desperdicio de recursos en el sistema de salud: el caso de la profesión médica y la enfermería en México. In: Knaul FM, Nigenda G, editores. Caleidoscopio de la Salud: de la investigación a las políticas y de las políticas a la acción. México, DF: Funsalud; 2003 [cited 2018 Oct 24]. p.125-34. Available from: http://funsalud.org.mx/portal/wp-content/uploads/2013/08/08-DesperdicioDeRecursos.pdf

[18] 18. Kjellsson G, Gerdtham UG. On correcting the concentration index for binary variables. J Health Econ. 2013;32(3):659-70. 10.1016/j.jhealeco.2012.10.012 23522656

[19] 19. Schneider MC, Castillo-Salgado C, Bacallao J, Loyola E, Mujica OJ, Vidaurre M, et al. Métodos de medición de las desigualdades de salud. Rev Panam Salud. 2002;12(6):398-415. 10.1590/s1020-49892002001200006 12690727

[20] 20. Secretaría de Salud (MEX), Dirección General de Información en Salud - DGIS. Cubos dinámicos: proyecciones CONAPO - versión Censo 2010. México, DF; 2018 [cited 2018 Oct 24]. Available from: http://www.dgis.salud.gob.mx/contenidos/basesdedatos/bdc_poblacion_gobmx.html

[21] 21. Secretaría de Gobernación (MEX), Consejo Nacional de Población Índices de Marginación por Entidad Federativa y Municipio 2010. México, DF: CONAPO; 2010 [cited 2018 Oct 24]. Available from: http://www.conapo.gob.mx/es/CONAPO/Indices_de_Marginacion_2010_por_entidad_federativa_y_municipio

[22] 22. Instituto Nacional de Estadística y Geografía. PIB por Entidad Federativa (PIBE): Base 2013. Aguascalientes (MEX): INEGI; 2018 [cited 2019 Dec 16]. Available from: https://www.inegi.org.mx/programas/pibent/2013/default.html#Documentacion

[23] 23. Institute for Health Metrics and Evaluation (IHME). Global Burden of Disease Study 2017 (GBD 2017) results. Seattle, WA: IHME; 2017 [cited 2020 Apr 5]. Available from: https://vizhub.healthdata.org/gbd-compare/

[24] 24. Organisation for Economic Co-operation and Development, OECD Library. Health at a Gance 2017: OECD indicators. Paris: OECD; 2017 [cited 2018 Oct]. Available from: https://read.oecd-ilibrary.org/social-issues-migration-health/health-at-a-glance-2017_health_glance-2017-en#page4

[25] 25. Nigenda G, Muños JA. Projections of specialist physicians in Mexico: a key element in planning human resources for health. Hum Resour Health. 2015;13(1):79. 10.1186/s12960-015-0061-z PMC4578266 26391878

[26] 26. Castillo-Laborde C. Human resources for health and burden of disease: an econometric approach. Hum Resour Health. 2011;9:4. 10.1186/1478-4491-9-4 PMC3039562 21269453

[27] 27. Organisation for Economic Co-operation and Development. OECD reviews of health systems: Mexico 2016. Paris: OECD Publishing; 2016 [cited 2019 Jan 18]. Available from: https://read.oecd-ilibrary.org/social-issues-migration-health/oecd-reviews-of-health-systems-mexico-2016_9789264230491-en#page1

[28] 28. Márquez M, Vazquez D, García C. Movilidad territorial en la formación de médicos en México. Salud Publica Mex. 1996;38(1):20-8. 8650592

[29] 29. Chen LC. Striking the right balance: health workforce retention in remote and rural areas. Bull World Heal Organ. 2010;88(5):323, A. 10.2471/BLT.10.078477 PMC2865673 20461215

[30] 30. Asociación Nacional de Universidades e Instituciones de Educación Superior. Información estadística de educación superior. México, DF: ANUIES; 2018 [cited 2020 Apr 5]. Available from: http://www.anuies.mx/informacion-y-servicios/informacion-estadistica-de-educacion-superior

[31] 31. Dal Poz MR, Kinfu Y, Kunjumen T. Counting health workers: definitions, data, methods and global results. Geneva: World Health Organization; 2007 [cited Apr 5]. Available from: https://www.who.int/hrh/documents/counting_health_workers.pdf

