# Supplementary material for: Socioeconomic factors and inequality in the distribution of physicians and nurses in Mexico
Source: Rev Saude Publica. 2020 Jun 3;54:58. doi: 10.11606/s1518-8787.2020054002011 (PMC7274212; doi:10.11606/s1518-8787.2020054002011)
Supplement: Translation

# Translation: Factores socioeconómicos y desigualdad en la distribución de médicos y enfermeras en México

**Authors:** Julio César Montañez-Hernández, Jacqueline Alcalde-Rabanal, Hortensia Reyes-Morales

**Affiliations:** I México Instituto Nacional de Salud Pública. Centro de Investigación en Sistemas de Salud. Cuernavaca, Mor, México

**Keywords:** Distribución de Médicos, Enfermeras y Enfermeros, provisión & distribución, Grupo de Atención al Paciente, Factores Socioeconómicos, Equidad en Salud

## Abstract

**OBJETIVO:**

Describir los recursos humanos en salud y analizar la desigualdad en su distribución en México.

**MÉTODOS:**

Estudio transversal basado en la Encuesta Nacional de Ocupación y Empleo del cuarto trimestre de 2018 en México. Se consideraron como recursos humanos en salud médicos y enfermeras con licenciatura, y personal de enfermería auxiliar/técnica con estudios concluidos. Se agrupó a los estados por grado de marginación y se estimó densidades de recursos humanos en salud por 1.000 habitantes, Índices de Disimilitud e Índices de Concentración (IC) como medidas de desigualdad en la distribución.

**RESULTADOS:**

La densidad de recursos humanos en salud fue de 4,6 por 1.000 habitantes; se observó heterogeneidad entre los estados con que van 2,3 hasta 10,5 por 1.000 habitantes. La desigualdad fue mayor en los estados con muy bajo grado de marginación (IC = 0,4) que en los estados de muy alto grado (IC = 0,1), y fue mayor la desigualdad en la distribución de los médicos (IC = 0,5) que en las enfermeras profesionales (IC = 0,3) entre los estados. Para que todos los estados tuvieran una densidad igual a la nacional de 4,6, se tendrían que distribuir alrededor de 12,6% de los recursos humanos en salud entre los estados que estuvieron por debajo de la densidad nacional. Adicionalmente, 17 estados tuvieron una densidad superior al umbral de 4,5 por 1.000 habitantes propuesto en la Estrategia Global en Recursos Humanos para la Salud. Eso implica un déficit de casi 60 mil recursos humanos en salud entre los 15 estados por debajo del umbral.

**CONCLUSIONES:**

En México existe desigualdad en la distribución de recursos humanos en salud, diferenciada en los estados. Mecanismos gubernamentales a través de una política de recursos humanos podrían incentivar el equilibrio en el mercado de laboral de los médicos y enfermeras.

## INTRODUCCIÓN

Los recursos humanos que laboran en las instituciones de salud son considerados uno de los pilares para el adecuado desempeño de los sistemas de salud^1,2^. Anand y Bärnighausen hacen una crítica al marco conceptual de los sistemas de salud propuesto por la Organización Mundial de la Salud (OMS) que considera a los recursos humanos en salud (RHS) como uno de los bloques del sistema^1^. Proponen un marco conceptual en el que los recursos humanos son el centro de los sistemas de salud^3^. El cumplimiento de las metas y la satisfacción de los pacientes dependen de los RHS, dado que son ellos quienes realizan las actividades de provisión de servicios preventivos y curativos, entregan información sobre el diagnóstico, tratamiento y seguimiento, y deciden qué tecnología y/o medicamento utilizar, por lo cual su tamaño, composición y distribución son muy relevantes para asegurar el acceso de la población a los servicios de salud^3^.

La disponibilidad de RHS, medida a través de la densidad por número de habitantes, ha sido relacionada con indicadores de cobertura. Se ha encontrado una relación positiva con algunas intervenciones como cobertura de vacunación o cobertura de partos atendidos por personal calificado^4^. En 2004, la Iniciativa Conjunta de Aprendizaje (Joint Learning Iniciative, [JLI]), recomendó una densidad de 2,5 por 1.000 habitantes para alcanzar una cobertura de 80,0% de inmunización contra el sarampión y partos atendidos por personal de salud^5^. En el marco de los Objetivos de Desarrollo del Milenio (ODM), el informe mundial de la OMS de 2006 sugirió un umbral mínimo de 2,3 médicos, enfermeras y parteras por cada mil habitantes para alcanzar un 80,0% de partos atendidos por personal calificado; 57 países no alcanzaban tal indicador^4^.

En 2016, en el marco de los Objetivos de Desarrollo Sostenible (ODS)^6^, la OMS estableció una densidad de 4,5 médicos, enfermeras y parteras por 1.000 habitantes como umbral mínimo requerido para la implementación de la Estrategia Global sobre Recursos Humanos para la Salud (EGRHS) en el marco de la cobertura universal de salud (CUS)^7^. A diferencia del indicador de 2006, este umbral contempla 12 indicadores relacionados con metas de la CUS y los ODS. Con este indicador, la OMS pronosticó que se necesitarían 18 millones más de trabajadores de la salud en los países de ingreso medio-bajo para alcanzar los ODS^7,8^, aunque se hizo enfasis en que este no debe representar una meta para todos los paises dadas sus diferencias en las necesidades de salud^7^.

En México, el gobierno federal lanzó en 2003 un seguro público de salud conocido como Seguro Popular de Salud (SPS) para brindar protección financiera a la población excluida de la seguridad social^9^, con un logro de afiliación de 53,5 millones de mexicanos hacia finales de 2017^10^. Adicionalmente, en el período 2005–2016, el gasto público en salud per cápita aumentó 31,0% y la densidad de médicos y enfermeras por cada mil habitantes se incrementó en 34,0 y 32,0%, respectivamente. A pesar de estos avances, el sistema público de salud enfrenta problemas de accesibilidad y calidad de atención, que pueden ser explicados por la carencia de una política explícita de recursos humanos en salud^11^, y por el desequilibrio entre las necesidades de salud de la población y los recursos humanos y financieros^11,12^.

A nivel nacional, la Secretaría del Trabajo y Previsión Social (STPS) realiza estimaciones periódicas del número de médicos y enfermeras con licenciatura que están empleados^13^; no obstante, estas estimaciones no muestran si los profesionales ejercen la profesión médica ni se especifica la duración de la jornada laboral. Otras fuentes de información como la Organización para la Cooperación y el Desarrollo Economicos (OCDE) reportaron para México una densidad de 2,4 y 2,9 médicos y enfermeras, respectivamente, en 2017. Sin embargo, estos datos incluyen a médicos internos (pre-grado) y residentes (en posgrado de especialización), utilizan datos provenientes de distintas fuentes de información y en ellas se reconoce la posibilidad de duplicación^14^. En ambos casos, el indicador de densidad de RHS puede estar sobreestimado y no es posible calcular el déficit de profesionales que son necesarios para alcanzar el umbral de 4,5 recomendado en la EGRHS. Por lo anterior, el presente estudio tuvo como objetivo describir los RHS y analizar la desigualdad en su distribución en México.

## MÉTODOS

Estudio transversal realizado a partir de análisis secundario de datos para describir características del personal médico y de enfermería. Se utilizó la Encuesta Nacional de Ocupación y Empleo (ENOE), realizada por el Instituto Nacional de Estadística y Geografía (INEGI). La ENOE tiene un diseño probabilístico, biétapico, estratificado y por conglomerados, se levanta trimestralmente, tiene cobertura geográfica nacional y estatal, y su objetivo es proporcionar información sobre las características ocupacionales de la población de 15 años y más^15^. Las bases de datos están disponibles públicamente y no contienen información identificable de los individuos.

Se construyeron indicadores de desigualdad con base en la ENOE del cuarto trimestre de 2018 (trimestre IV-2018). Todas las personas con estudios en medicina o enfermería que refirieron haber terminado sus estudios de licenciatura al momento de la encuesta fueron consideradas Recursos Humanos en Salud (RHS) o personal sanitario. También se incluyeron a quienes estudiaron enfermería a nivel técnico (enfermería auxiliar).

### Variables16,17

Personal empleado/ocupado (Emp): personal sanitario que trabajaba 20 horas o más a la semana realizando funciones de acuerdo a su formación acádemica en el área de la salud, o realizaba funciones administrativas en el sector salud.

Personal desempleado (Des): personal sanitario que en la semana de referencia de la encuesta buscó trabajo porque no estaba vinculado a una actividad económica.

Personal con subempleo cuantitativo (SubCt): personal sanitario empleado/ocupado menos de 20 horas a la semana y con funciones de acuerdo a su profesión, o bien, que ejercía la profesión sanitaria como un empleo secundario.

Personal con subempleo cualitativo (SubCl): personal sanitario empleado/ocupado pero con funciones o actividades ajenas a su formación acádemica, independientemente de las horas laborales por semana.

Población Económicamente Activa (PEA): personal sanitario empleado, subempleado y desempleado.

### Medidas de Desigualdad

Densidades de Recursos Humanos en Salud por 1.000 habitantes:

a.Densidad de recursos humanos empleados (DRHS): $\frac{Emp}{Población\;total}\;x\;1.000$.

b.Densidad de recursos humanos PEA: $\frac{Emp\;+\;SubCt\;+\;SubCl\;+\;Des}{Población\;total}\;x\;1.000$.

En ambos casos, se obtuvo el déficit de RHS respecto al umbral recomendado en la EGRHS de acuerdo con las siguientes fórmulas:

c.Déficit de empleados: $\frac{(4,5–\mathrm{Densidad}\;\mathrm{de}\;\mathrm{recursos}\;\mathrm{humanos}\;\mathrm{empleados})\;x\;\mathrm{población}\;\mathrm{total}}{1.000}\;$.

d.Déficit de PEA: $\frac{(4,5–\mathrm{Densidad}\;\mathrm{de}\;\mathrm{recursos}\;\mathrm{humanos}\;\mathrm{PEA})\;x\;\mathrm{población}\;\mathrm{total}}{1.000}\;$.

Se analizaron los factores socioecónomicos (edad, escolaridad, localidad de residencia y características de empleo) en ambas profesiones, y se calcularon las densidades de recursos humanos en salud por entidad federativa de 1) personal sanitario empleado por 1.000 habitantes (DRHS), y 2) población económicamente activa (PEA) por 1.000 habitantes. En ambos casos, se compararon las densidades con el umbral de 4,5 por 1.000 recomendado en la EGRHS y se estimó el número de trabajadores de la salud necesarios para alcanzar dicho umbral.

### Índice de disimilitud

El índice de disimilitud (ID) es uno de los indicadores usados en el análisis de las desigualdades en salud^18,19^. Su cálculo proviene de la fórmula $\sum_{j=1}^{32}\;\frac{1}{2}\;\left|s_{jh}\;-\;s_{jp}\right|$, donde *s*_*jh*_ es la proporción del personal sanitario en el estado *j* respecto al total nacional, y *s*_*jp*_ es la proporción de la población en el estado *j* respecto a la población nacional. En este estudio, el ID se interpretó como la proporción (o porcentaje) de trabajadores que tendrían que redistribuirse entre los estados para que todos tuvieran la misma DRHS.

### Índice y curva de concentración

Otro índice usado en el análisis de las desigualdades en salud es el índice que proviene de la Curva Concentración (CC)^18,19^. En este estudio, se construyeron las curvas considerando a los estados como unidad de análisis. Se calculó la proporción de RHS en el estado *j* respecto al total nacional (*s*_*jh*_), y se calculó la proporción de los Años de Vida Saludable perdidos por Discapacidad (AVISA) en el estado *j* respecto al total nacional (*s*_*jd*_). La curva de concentración grafica la proporción *acumulada* de AVISA por los estados (empezando con el estado con la menor proporción de AVISA en el eje de las ordenadas, y finalizando con la mayor) con las proporciones *acumuladas* de RHS. Si los trabajadores están igualmente distribuidos entre los estados, de acuerdo con su porcentaje de la carga de la enfermedad nacional, la curva de concentración coincide con la diagonal que une los puntos (0,0) y (1,1), llamada línea de igualdad. El área entre la CC y la línea de igualdad es el Índice de Concentración (IC) y entre mayor sea el valor absoluto del IC más grande es la desigualdad. Se construyó un gráfico de dispersión entre la DRHS por 1.000 habitantes y los AVISA por 100 mil habitantes en cada estado^18^. Adicionalmente, se construyó otro gráfico de dispersión entre la DRHS por 1.000 habitantes y el Producto Interno Bruto (PIB) *per cápita,* y se estimó la correlación de Spearman.

### Análisis

Se utilizaron los tres indicadores de desigualdad en la distribución de recursos humanos a nivel nacional y por estados agrupados por grado de marginación: la DRHS por 1.000 habitantes, el ID y el IC. La información del tamaño de la población proviene de las proyecciones de población del Consejo Nacional de Población (CONAPO) disponibles en la página electrónica de la Dirección General de Información en Salud (DGIS) de la Secretaría de Salud^20^. La clasificación y agrupación de los estados según grado de marginación proviene de la clasificación propuesta por CONAPO^21^. Los datos del Producto Interno Bruto (PIB) se obtuvieron de la página electrónica del Instituto Nacional de Estadística y Geografía (INEGI)^22^. La información de los AVISA se obtuvo de las estimaciones de la Carga Global de la Enfermedad realizadas por el Instituto de Métricas y Evaluación de la Salud de 2017 (IHME, en inglés)^23^.

Los análisis se realizaron utilizando los factores de expansión de la encuesta y se consideró su diseño complejo utilizando el módulo SVY del paquete STATA MP 13.0. Pruebas Chi-cuadrada de Pearson evalúan las diferencias en la distribución entre el tipo de profesión (medicina o enfermería) y sus factores socioeconómicos considerando p < 0.05 para la significancia estadística e intervalos de confianza al 95% (IC95%).

## RESULTADOS

En 2018, había 413 mil médicos y 714 mil enfermeras en México, de los cuales 62,9% y 44,4%, respectivamente, estaban empleados 20 horas o más en el sector salud (Tabla 1), equivalente a 260,482 médicos y 317,280 enfermeras (Tabla 2); 24,8% de los médicos [IC95% 20,7–29,3] y 1.5% de las enfermeras tenían una especialidad/posgrado [IC95% 1,0–2,2]. Por el contrario, 46,8% [IC95% 44,1–49,6] eran enfermeras técnicos/auxiliares. Encontramos diferencias estadísticamente significativas en la distribución por edad y por características de empleo en ambas profesiones. En particular, el subempleo cualitativo fue mayor en el personal de enfermería (17,6%, [IC95% 15,3–20,2]) que en el personal médico (8,8%, [IC95% 6,9–11,0]), al igual que el desempleo (1,9%, [IC95% 1,2–2,9]; y 1,3%, [IC95%: 0,7–2,4], respectivamente) (Tabla 1).

**Tabla 1:** Factores socioeconómicos del personal médico y de enfermería. Encuesta Nacional de Ocupación y Empleo, ENOE, México, Trimestre IV, 2018.

	Total	Personal médico	Personal de enfermería
N	1.128.668	413.866	714.802
n	4.022	1.443	2.579
%	100%	36,7%	63,3%
	% [IC 95%]		
**Grupo de edad (años)**			
20-29	21,1 [19,3–23,0]	16,6 [13,8–19,8]	23,7 [21,5–26,0]^a^
30-39	24,4 [22,2–26,8]	26,5 [22,3–31,1]	23,2 [20,7–25,9]
40-49	17,5 [15,7–19,4]	14,0 [11,4–17,0]	19,5 [17,2–22,0]
50-59	18,8 [16,8–21,0]	15,8 [12,8–19,3]	20,6 [18,1–23,4]
60-69	13,0 [11,2–15,0]	19,0 [15,5–23,1]	9,5 [7,8–11,4]
70+	5,2 [4,1–6,5]	8,1 [5,9–11]	3,5 [2,6–4,7]
**Escolaridad**			
Técnico/auxiliar	29,7 [27,4–32,0]		46,8 [44,1–49,6]^a^
Licenciatura	60,3 [57,7–62,9]	75,2 [70,7–79,3]	51,7 [48,9–54,5]
Especialidad/posgrado	10,0 [8,4–11,9]	24,8 [20,7–29,3]	1,5 [1,0–2,2]
**Estado civil**			
Sin pareja^b^	40,6 [38,1–43,1]	40,0 [35,8–44,3]	40,9 [38,0–44,0]^a^
Con pareja^c^	59,4 [56,9–61,9]	60,0 [55,7–64,2]	59,1 [56,0–62,0]
**Características de empleo**			
PEA			
Empleo	51,2 [48,6–53,8]	62,9 [58,8–66,9]	44,4 [41,3–47,5]^a^
Subempleo cuantitativo	4,2 [3,3–5,3]	5,7 [4,0–8,0]	3,3 [2,4–4,5]
Subempleo cualitativo	14,3 [12,7–16,2]	8,8 [6,9–11,0]	17,6 [15,3–20,2]
Desempleo	1,7 [1,2–2,4]	1,3 [0,7–2,4]	1,9 [1,2–2,9]
PNEA^d^	28,6 [26,3–31,0]	21,3 [18,1–24,8]	32,8 [29,9–35,9]
**Tamaño de localidad**			
Rural	6,1 [5,6–6,8]	4,7 [4,3–5,1]	7,0 [6,4–7,7]
Urbano	93,9 [93,2–94,4]	95,3 [94,9–95,7]	93[92,3–93,6]
**Grado de marginación**^e^			
Muy baja	24,3 [23,0–25,7]	27,3 [24,8–30,0]	22,6 [21,3–24,0]^a^
Baja	32,0 [30,8–33,2]	31,1 [29,3–33,1]	32,4 [31,1–33,7]
Media	14,2 [13,6–14,9]	13,6 [12,6–14,6]	14,6 [13,9–15,3]
Alta	21,7 [20,7–22,8]	20,0 [18,9–21,1]	22,7 [21,5–23,9]
Muy alta	7,8 [7,3–8,4]	8,0 [7,3–8,7]	7,7 [7,1–8,3]

ENOE: [Encuesta Nacional de Ocupación y Empleo]; PEA: [Población Económicamente Activa]; PNEA [Población No Económicamente Activa]

^a^ p < 0,000; ^b^ Incluye solteros, divorciados y viudos; ^c^ Incluye unidos y casados; ^d^ Población No Económicamente Activa: incluye personas que se dedican a actividades del hogar, estudiantes, pensionados y discapacitados. ^e^ **Grado de marginación**: Ciudad de México ,Coahuila, Baja California, Nuevo León (**Muy bajo**); Aguascalientes, Baja California Sur, Colima, Chihuahua, Jalisco, Estado de México, Tamaulipas (**Bajo**); Durango, Guanajuato, Morelos, Nayarit, Querétaro, Quintana Roo, Sonora, Tlaxcala, Zacatecas (**Medio**); Campeche, Hidalgo, Michoacán, Puebla, San Luis Potosí, Sinaloa, Tabasco, Veracruz, Yucatán (**Alto**); Chiapas, Guerrero, Oaxaca (**Muy alto**).

**Tabla 2:** Medidas de desigualdad en la distribución del personal médico y de enfermería empleado (DRHS) en estados agrupados por grado de marginación: Densidad por 1.000 habitantes (DRHS), Índice de Disimilitud (ID) e Índice de Concentración (IC). Encuesta Nacional de Ocupación y Empleo, ENOE, Mexico, Trimestre IV, 2018.

	Nacional (32)	Grado de marginación (número de estados)				

		Muy bajo (4)	Bajo (7)	Medio (9)	Alto (9)	Muy alto (3)
Población (miles)	124.738	20.786	39.261	20.849	30.687	13.155
Total de RHS (medicina y enfermería)	577.762	150.807	173.821	83.027	124.337	45.770
DRHS	4,6	7,3	4,4	4,0	4,0	3,5
ID	12,6	18,9	6,6	7,7	11,5	2,7
IC	0,4	0,4	0,5	0,2	0,2	0,1
Medicina	260.482	80.511	77.279	33.587	49.861	19.244
DRHS	2,1	3,9	2,0	1,6	1,6	1,5
ID	16,3	30,0	6,5	7,1	10,6	5,5
IC	0,5	0,5	0,5	0,3	0,2	0,0
Enfermería técnica	111.856	25.811	46.507	11.562	25.051	2.925
DRHS	0,9	1,2	1,2	0,5	0,8	0,2
ID	25,4	8,6	9,4	13,4	33,6	29,0
IC	0,5	0,3	0,6	0,3	0,2	0,2
Enfermería profesional	205.424	44.485	50.035	37878	49.425	23.601
DRHS	1,6	2,1	1,3	1,8	1,6	1,8
ID	18,2	12,1	26,1	12,4	15,4	6,1
IC	0,3	0,3	0,3	0,2	0,2	0,1

DRHS: Densidad de Recursos Humanos en Salud empleados por 1.000 habitantes. ID: Índice de Disimilitud. IC: Índice de Concentración.

La densidad de profesionales de la salud (DRHS) por 1.000 habitantes fue de 4,6 (2,1 en personal médico, 0,9 y 1,6 en personal de enfermería técnica y profesional, respectivamente), superando el umbral de 4,5 recomendado en la EGRHS (Tabla 2). Sin embargo, la brecha entre la densidad actual y el umbral de 4,5 fue heterogénea entre los estados agrupados por grado de marginación: mientras en los estados de muy bajo grado la densidad es de 7,3 por mil habitantes, en el grupo de muy alta marginación fue de 3,5 por 1.000 habitantes. El ID señaló que, para que en todos los estados exista una densidad de 4,6 por 1.000 habitantes, tendrían que redistribuirse 12,6% del total del personal sanitario, mientras que en el grupo de estados de muy alta marginación se tendrían que distribuir 2,7% para que todos tuvieran una densidad de 3,5 por 1.000 habitantes. Asimismo, el IC nacional (0,4) indicó desigualdad similar a nivel nacional que al interior de los grupos de muy bajo y bajo grado de marginación (0,4 y 0,5, respectivamente), y la desigualdad fue similar en los grupos de grado medio y alto (0,2 y 0,2, respectivamente) aunque menor que en los grupos anteriores.

La mitad de los estados (50,0%) con las menores proporciones de AVISA concentraron menos de 25,0% de toda la fuerza laboral, mientras que cinco de ellos (Nuevo León, Puebla, Jalisco, México y Ciudad de México) concentraron más de 40,0% (CI = 0,4). Este patrón fue originado principalmente por la desigualdad en la distribución del personal médico (CI = 0,5) y de enfermería técnica (CI = 0,5), y en menor medida a la distribución de la enfermería profesional (CI = 0,3) (Figura 1). Adicionalmente, no se observó una correspondencia entre la DRHS por 1.000 habitantes en cada estado y su tasa de AVISA por 100 mil habitantes; Veracruz y Ciudad de México tuvieron tasas similares pero mostraron la más baja y más alta densidad DRHS, respectivamente. Por el contrario, Chihuahua y Sinaloa tuvieron densidades DRHS por encima de 4,5 pero mostraron la más alta y más baja tasa de AVISA, respectivamente (Figura 2a). Por otro lado, existió una correlación positiva entre la DRHS por 1.000 habitantes y el Producto Interno Bruto (PIB) *per cápita* (rho = 0,3718, p < 0,05). Esto coincidió con lo descrito anteriomente, según lo cual los grupos de entidades de muy baja y muy alta marginación tenían mayores y menores DRHS, respectivamente (Figura 2b).

**Figura 1:** Curva de concentración de los Recursos Humanos en Salud (RHS) empleados entre los estados ordenados de acuerdo a la proporción de Años de Vida Saludable perdidos por Discapacidad, AVISA. Encuesta Nacional de Ocupación y Empleo, ENOE, México, Trimestre IV, 2018. Aguascalientes (AGS), Baja California (BC), Baja California Sur (BCS), Campeche (CAMP), Coahuila (COAH), Colima (COL), Chiapas (CHIS), Chihuahua (CHIH), Ciudad de México (CDMX), Durango (DGO), Guanajuato (GTO), Guerrero (GRO), Hidalgo (HGO), Jalisco (JAL), Estado de México (MEX), Michoacán (MICH), Morelos (MOR), Nayarit (NAY), Nuevo León (NL), Oaxaca (OAX), Puebla (PUE), Querétaro (QRO), Quintana Roo (QROO), San Luis Potosí (SLP), Sinaloa (SIN), Sonora (SON), Tabasco (TAB), Tamaulipas (TAMP), Tlaxcala (TLAX), Veracruz (VER), Yucatán (YUC), Zacatecas (ZAC).

**Figura 2:** Dispersión entre la densidad de Recursos Humanos en Salud (RHS) empleados con las tasas de Años de Vida Saludable perdidos por Discapacidad (AVISA) por 100,000 habitantes y con el PIB *per capita*. Encuesta Nacional de Ocupación y Empleo, ENOE, Mexico, Trimestre IV, 2018. Aguascalientes (01), Baja California (02), Baja California Sur (03), Campeche (04),Coahuila (05), Colima (06), Chiapas (07), Chihuahua (08), Ciudad de México (09), Durango (10), Guanajuato (11), Guerrero (12), Hidalgo (13), Jalisco (14), Estado de México (15), Michoacán (16), Morelos (17), Nayarit (18), Nuevo León (19), Oaxaca (20),Puebla (21), Querétaro (22), Quintana Roo (23), San Luis Potosí (24), Sinaloa (25), Sonora (26), Tabasco (27), Tamaulipas (28),Tlaxcala (29),Veracruz (30), Yucatán (31), Zacatecas (32).

Diecisiete entidades alcanzaron una densidad de 4,5 (Figuras 2, 3a y 3b). La estimación de la brecha en número de trabajadores para alcanzar el umbral muestra que se requirirían 59.618 trabajadores empleados 20 horas o más por semana en el sector salud para que los 15 estados restantes alcanzaran dicho umbral. Sin embargo, en solo cuatro de ellos (Guanajuato, Chiapas, Veracruz y Estado de México) la brecha osciló entre 4.332 y 17.552 (Figura 3b). Por otro lado, en el escenario en que toda la población economicamente activa (PEA) de RHS estuviera empleada 20 o más horas en el sector salud, la densidad nacional sería 6,5 aunque todavía tres estados no alcanzarían el umbral (Figuras 3c y 3d).

**Figura 3:** Densidad de Recursos Humanos en Salud (RHS): empleados y Población Económicamente Activa (PEA) por 1.000 habitantes, y sus déficit para alcanzar el umbral de la Estrategia Global de Recursos Humanos en Salud (EGRHS). Encuesta Nacional de Ocupación y Empleo, ENOE, Mexico, Trimestre IV, 2018. Aguascalientes (01), Baja California (02), Baja California Sur (03), Campeche (04),Coahuila (05), Colima (06), Chiapas (07), Chihuahua (08), Ciudad de México (09), Durango (10), Guanajuato (11), Guerrero (12), Hidalgo (13), Jalisco (14), Estado de México (15), Michoacán (16), Morelos (17), Nayarit (18), Nuevo León (19), Oaxaca (20),Puebla (21), Querétaro (22), Quintana Roo (23), San Luis Potosí (24), Sinaloa (25), Sonora (26), Tabasco (27), Tamaulipas (28),Tlaxcala (29),Veracruz (30), Yucatán (31), Zacatecas (32).

## DISCUSIÓN

Nuestro estudio muestra una desventaja de México en la disponibilidad de RHS respecto al promedio de los países pertenecientes a la OCDE^24^. Si bien, de acuerdo a los criterios de la EGRHS, la densidad de trabajadores de la salud podría considerarse aceptable, la desigualdad al interior del país se refleja en la variabilidad entre estados, en particular aquellos con alta marginación que no alcanzan el umbral. Si se reconoce que en los datos de la OCDE se incluye a los médicos internos y residentes, y existe la posibilidad de doble conteo de médicos y enfermeras que trabajan simultáneamente en el sector público y privado, la disponibilidad de RHS para las áreas con menor densidad probablemente quedará por debajo de la demanda actual y con mayor déficit para años futuros. En este sentido, proyecciones de requerimiento de médicos especialistas en México han estimado que las necesidades de salud derivadas del envejecimiento poblacional requerirán mayor número de especialistas en medicina interna y cirugía que en pediatría, lo que implicará un reto creciente para la provisión de servicios^25^.

Un hallazgo relevante fue la falta de consistencia en la relación entre la densidad de trabajadores de la salud y las tasas de AVISA. Se esperaría una correlación negativa, como ha sido documentado en otros estudios, que apoyaría la evidencia de contar con una mayor cantidad de profesionales de la salud para disminuir la carga de enfermedad^26^. Dicho resultado pudiera reflejar otras áreas débiles del propio sistema de salud, como insuficiencia en equipamiento o insumos requeridos, deficiencia en la calidad de la atención, así como condiciones propias de la transición demográfica y epidemiológica. Eso resulta en necesidades de salud crónico-degenerativas que derivan en discapacidad para una mayor proporción de la población^27^.

En contraste, la relación positiva entre la DRHS y el PIB estatal podría ser explicada por las siguientes razones: 1) el personal médico calificado puede cambiar su residencia a las regiones de México con ciudades identificadas como focos de atracción en la movilidad médica^28^, que tienen mayor desarrollo económico y brindan mayores oportunidades laborales y expectativas de mejor ingreso^29^; o bien, 2) la distribución del número de matrículas registradas en las universidades a lo largo del país donde seis estados, de marginación muy baja o baja, concentran al 50 y 35,0% de las matrículas de estudiantes de medicina y enfermería en el ciclo escolar 2017–2018, respectivamente, y tres entidades de muy alta marginación concentran al 15,7% de los estudiantes de enfermería matriculados y 6,5% de los estudiantes de medicina matriculados^30^. Estos desequilibrios en la distribución de los recursos humanos representan un reto para el sistema de salud, ya que la población que vive en las zonas de alto grado de marginación presenta menor posibilidad en el acceso a los servicios de salud.

Son limitaciones de este análisis la dificultad para identificar la movilidad laboral, que puede afectar la estimación de las densidades estatales en el tiempo, y limitaciones provenientes de la ENOE: a) no es posible distinguir entre niveles de atención ni entre esquemas de aseguramiento, lo que impide establecer de manera más precisa desigualdades entre grupos poblacionales o necesidades de salud; b) no se incluyó a las parteras debido a que no es posible identificarlas en la encuesta, y tampoco se incluyeron otras categorías de personal de salud como dentistas, farmacéuticos, técnicos de laboratorio o promotores comunitarios que también son considerados como fuerza laboral en salud^31^, pero que no se consideran en la construcción del umbral de 4,5 por 1.000 habitantes; c) la construcción de la tasas de empleo no contempla la posibilidad de que médicos y enfermeras pudieran dedicarse a actividades de docencia e investigación en salud, por lo que es muy posible que existan profesionales que son profesores e investigadores que estén incluidos en la categoría de personal subempleado, y 5) la ENOE es una encuesta representativa a nivel nacional y estatal de toda la población mayor de 15 años; el personal médico y de enfermería representa menos de 2,0% de la población, lo que podría interpretarse como una debilidad de los resultados a nivel estatal. Sin embargo, dado el diseño aleatorio de la ENOE y que los tamaños de muestra son grandes para ambas profesiones, se puede asumir representatividad para este subgrupo.

Los resultados indican desigualdad en la distribución de los RHS a través de los estados, la cual puede estar potencialmente ligada al número de matrículas que se ofertan en las instituciones educativas, la preferencia del personal de salud para insertarse laboralmente en áreas con mejores condiciones de vida, y mayor disponibilidad de fuentes de trabajo en estados con mayor infraestructura en salud. Es prioritario el establecimiento de una nueva política de recursos humanos que, a partir de las necesidades de salud de la población, articule la formación de médicos, enfermeras y otros profesionales de la salud para su incorporación en las instituciones de salud considerando las áreas de mayor demanda. Además, es necesaria la regulación de la práctica profesional, que incentive el equilibrio en el mercado de trabajo público y privado.
